# Image-Based Ship Detection Using Deep Variational Information Bottleneck

**DOI:** 10.3390/s23198093

**Published:** 2023-09-26

**Authors:** Duc-Dat Ngo, Van-Linh Vo, Tri Nguyen, Manh-Hung Nguyen, My-Ha Le

**Affiliations:** 1 Faculty of Electrical and Electronics Engineering, University of Technology and Education, Ho Chi Minh City 7000, Vietnam; datnd.ncs@hcmute.edu.vn (D.-D.N.); 20139080@student.hcmute.edu.vn (V.-L.V.); 2 Faculty of Information Technology, Industrial University of Ho Chi Minh City, Ho Chi Minh City 7000, Vietnam; tringuyen21072002@gmail.com

**Keywords:** ship detection, maritime security, information bottleneck

## Abstract

Image-based ship detection is a critical function in maritime security. However, lacking high-quality training datasets makes it challenging to train a robust supervision deep learning model. Conventional methods use data augmentation to increase training samples. This approach is not robust because the data augmentation may not present a complex background or occlusion well. This paper proposes to use an information bottleneck and a reparameterization trick to address the challenge. The information bottleneck learns features that focus only on the object and eliminate all backgrounds. It helps to avoid background variance. In addition, the reparameterization introduces uncertainty during the training phase. It helps to learn more robust detectors. Comprehensive experiments show that the proposed method outperforms conventional methods on Seaship datasets, especially when the number of training samples is small. In addition, this paper discusses how to integrate the information bottleneck and the reparameterization into well-known object detection frameworks efficiently.

## 1. Introduction

Detecting and tracking vessels are routine and pressing tasks due to considerations related to security, safety, and environmental management. The regulation V/19-1 of the 1974 SOLAS convention requires specific ships must be equipped with a Long-Range Identification and Tracking (LRIT) system [1]. However, the information is only transmitted automatically every six hours from the LRIT equipment installed on the ship via the Inmarsat satellite. Another solution for tracking and identifying ships is the automatic identification system (AIS) [2]. An automatic identification system (AIS) is an automated tracking system that displays other vessels in the vicinity. The broadcast transponder system operates in the VHF mobile maritime band; hence, the transceiver range is limited and depends on weather conditions. Moreover, the system can be turned off manually and can be easily tampered with. For the aforementioned reasons, each country has established a radar-based monitoring system [3] for overseeing its maritime regions. This system is robust in various environments and can detect multiple objects at far distances. However, the system can provide only the distance and bearing of the object, not information about each vessel category. Modern naval observation stations have recently been equipped with high optical zoom cameras to supplement the traditional radar system. These systems can support day and night vision with outstanding image quality. This allows using image information for maritime border security.

Detecting and categorizing ships from images are applications of object detection [4] in computer vision. Recently, deep learning is the most successful solution for these applications by training a detector. The early deep-learning-based detectors RCNN [5] or FastRCNN [6] have a better accuracy compared with hand-crafted detectors [7], but they are not efficient enough to work in real-time. Later, one-stage detectors SDD [8] or YOLO [9] have been introduced to speed up the inference. These methods need anchor boxes and post-processing to detect objects. Recently, feature pyramid networks [10] and decoupled head [11] have been introduced to detect small objects with higher performance. In addition, the success of transformer [12] on natural language processing (NLP) also opens a new approach for object detection when transformer-based detectors [13,14] could obtain a comparable result with CNN-based detectors [8,11]. Transformer-based detectors do not require anchor boxes and post-processing for detection. Hence, it can work as an end-to-end training process.

Deep-learning-based detectors have reported many promising results. However, training a ship detector for maritime security is still an open question with several challenges. First, high-quality datasets may not be available for research. Satellite datasets such as [15,16,17] can provide many images for ship detection; however, cameras at naval observation stations are front-view cameras. The PASCAL VOC2012 database [18] provides front-view ship images, but the number of training samples is very limited. In the dataset, few ship objects are available, and all of them are categorized as a single “boat” class. Recently, a large-scale dataset [19] has been introduced. This dataset includes 31,455 images with six classes. However, only 7000 images are published for research purposes. The second challenge in ship detection is the complexity of environmental factors. The large-scale dataset [19] had reported several factors such as background selection, lighting environment, visible proportion, and occlusion have been reported. These above challenges raise a research question: Could we learn generalization features that focus on a ship and eliminate environmental factors based on small datasets?

Given a limited dataset, conventional methods use data argumentation to address these challenges by enriching the training dataset. In classification tasks, well-known data argumentation are listed as flipping, rotation, scaling, cropping, translation, and adding Gaussian noise have been integrated into training frameworks. In object detection tasks, Mosaic is a successful solution that has been introduced in YOLOv4 [20] and reused in later YOLO versions. However, these data argumentations may not model environmental factors such as background selection and visible proportion in an ocean scenario. Recently, Light_SDNet [21] enriched a large-scale ship dataset [19] by adding haze and rain to the original ship images.

**Figure 1:**
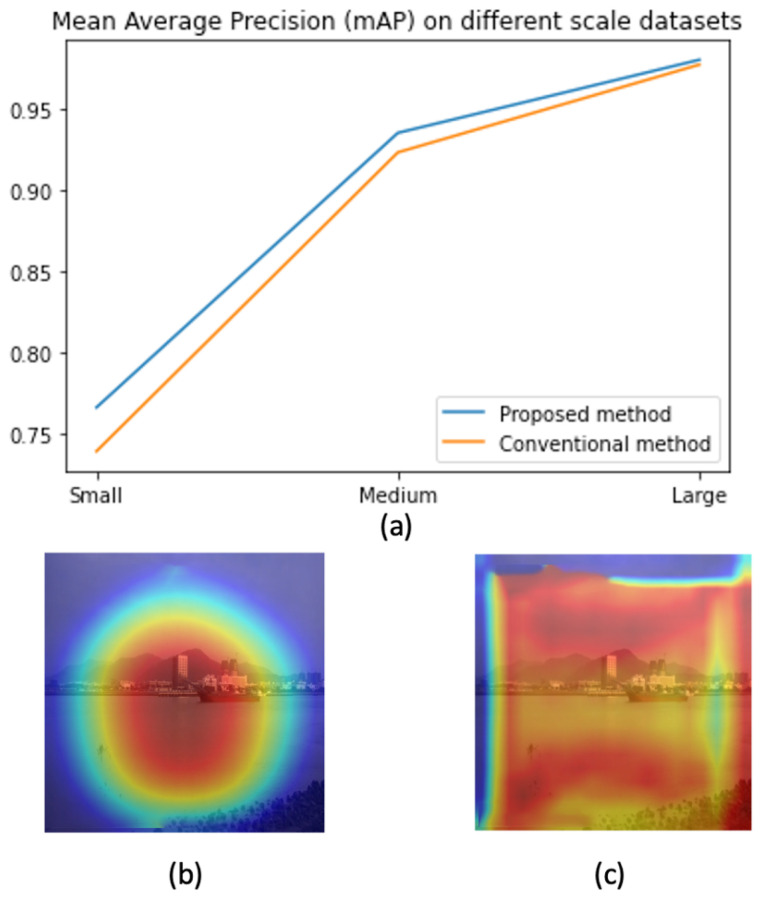
The contribution of the proposed method. (**a**) Performance on different scale datasets (**b**) A heat map by the proposed method (**c**) A heat map by a baseline method.

In this paper, we address the challenge by co-designing two factors. First, we aim to learn features that focus only on ships and skip all background information. This will help to address the background selection challenges. Second, a reparameterization is used to enrich the dataset in feature space. Unlike conventional methods where the training data are increased in the image domain, we aim to add some uncertainty in the feature domain. Hence, the classifier can work with noise from the environment. The variational information bottleneck (VIB) [22] is used to select features that focus only on objects and eliminate background information. Later, these features add some uncertainty before feeding to a classification head. This approach could be integrated into any well-known detectors; however, this paper uses YOLOX [11] as our baseline due to its outperforming on testing datasets. As shown in Figure 1, the proposed method help to have a better performance, especially on small datasets. Also, heatmaps in Figure 1 prove that learned features focus on objects. It means the model may work better in a practical environment.

Conventionally, a detector includes a backbone, a neck, and a decoupled head that addresses regression and classification tasks. Given an input image *x*, the backbone extracts the feature $F^{i}\left(x\right)$ at different scales *i*th. For each feature $F^{i}\left(x\right)$, the neck module connects the feature to the corresponding classification head and regression head as in YOLOX [11]. We found that box regression is a critical task to ensure the success of a detector in training; hence, the regression head in [11] is reused to make the network converge smoothly. The modification is on the classification head. On this head, an encoder extracts the mean $\mu^{i}\in R^{dW^{i}H^{i}}$, and the corresponding variance $\sigma^{i}\in R^{dW^{i}H^{i}}$ of features $F^{i}\left(x\right)$. As the classifier must work well with variant features, we sample latent features $z^{i}∼N\left(\mu^{i},\;\sigma^{i}\right)$ from the mean and variance. Then, the classification result $y^{i}\in R^{KW^{i}H^{i}}$ is predicted from sampled feature $z^{i}$ by $y^{i}=cls\left(z^{i};\theta^{i}\right)$. A good feature *z* must represent fine-grained features of ship categories. Hence, the mutual information [23] I(y;z) should be maximized. Additionally, the background information from input *x* should be eliminated on feature space *z*. Hence, the mutual information I(x;z) should be minimized. Two constraints are optimized together in a variational information bottleneck (VIB) loss [22]. Since this loss could be integrated into any supervised learning framework, it could be accompanied by regression and object losses to train a detector.

In summary, the paper’s contributions are listed below:

1. Regularly, VIB and the parameterization trick are used in classification tasks. However, this paper discusses integrating these techniques into object-detection frameworks. The method outperforms SoTA in detecting ship objects, especially in small-scale datasets.
2. We carefully test the effect of VIB and parameterization at different positions in decoupled heads. The result shows that VIB can only work on the classification head and should not allow the VIB loss effect on the regression head.
3. A feature analysis proves that the proposed method could learn feature focus on objects rather than the background.

## 2. Related Works

### 2.1. Object Detection

Deep learning-based object detection has been developed rapidly in recent years. Early works [5,6,24,25,26] are considered as two-stage detectors because two processes are needed in an inference. First, a region that may involve an object is selected. The region is considered the location of an object on the image. Second, the region is cropped and fed to a classifier to estimate its categories. To detect small objects, the feature pyramid [10] had been used to extract features in multiple levels. The FPN has two pathways: a bottom-up pathway which is a ConvNet computing feature hierarchy at several scales, and a top-down pathway that upsamples coarse feature maps from higher levels into high-resolution features. FPN is a region proposal network (RPN) in Faster R-CNN [25].

Another approach to solving the detection is single-state detectors [8,9,27,28,29,30,31]. These single-stage detectors directly predict the image pixels as objects and their bounding box attributes. YOLO [9] is the first representation of a single-stage detector. It can work very fast, but the accuracy is not high. Single Shot MultiBox Detector (SSD) [8] was the first method of single-stage detectors that matched the accuracy of contemporary two-stage detectors like Faster R-CNN [25]. RetinaNet [30] proposed a focal loss as the means to remedy the imbalance between background and foreground objects. The focal loss parameter reduces the loss contribution from easy examples. The authors demonstrate its efficacy with the help of a simple, single-stage detector. Later, CenterNet [29] models objects as points. As the predictions are points but not bounding boxes, non-maximum suppression (NMS) [9] is not required for post-processing. EfficientDet [31] introduces efficient multi-scale features (BiFPN) and model scaling. BiFPN is a bi-directional feature pyramid network with learnable weights for cross-connection input features at different scales. In addition, it jointly scales up all dimensions of the backbone network, BiFPN network, class/box network, and resolution. Therefore, this method achieves better efficiency and accuracy than previous detectors while being smaller and computationally cheaper.

The next generation of the YOLO family, such as YOLOv4 [20], and YOLOv5 [32], incorporated many exciting ideas to design a fast object detector that could work in existing production systems. Recently, YOLOX [11] has introduced the decouple head to separate the classification and regression tasks. It allows the detector to convert easily. Also, data arguments like Mosaic and Mixup have been introduced to increase accuracy.

Transformer [12] had been very successful in NLP [33]. Therefore, many works [13,14,34] have tried to apply the transformer concept to object detection. Transformers present a paradigm shift from CNN-based neural networks. While its application in vision is still nascent, its potential to replace convolution from these tasks is very real. The state-of-the-art transformer-based detectors have promising results on the COCO dataset [35], but utilize comparatively higher parameters than convolutional models.

### 2.2. Ship Detection

Several modifications of well-known object detection methods have been introduced to improve the performance of ship detectors. Liu_2022 [36] based on the SSD [8] framework and VGG backbone to detect a ship on small scales. The author [36] used a local attention network to fuse cross-features; also, a merge module combines features from different scales to improve detection results. The YOLO family is also used by many works to enhance the detection of ship datasets. Based on the YOLO framework, Biaohua_2022 [37] introduced a “Cross-level Attention and Ratio Consistency Network” (CARC) for ship detection. In this paper, the backbone was Resnet-34; the neck was a cross-level-attention module that used channel attention and spatial attention to extract features at different scales. The features were concatenated and fed to a head. Cui_2019 [38], Liu_2020 [39], and Li_2021 [40] based on YOLOV3 to detect ships. Cui_2019 [38] introduced YOLOv3-ship consisting of dimension clusters, network improvement, and Squeeze-and-Excitation(SE) module embedding. Liu_2020 [39] introduced two new anchor-setting methods and cross-feature fusion to enhance the performance of YOLOV3. Instead of using the FPN [10] to connect the backbone to a head, the method used a Cross PANet, which can combine the location information of the low-level feature maps with the semantic information of the high-level feature maps. Li_2021 [40] is based on YOLOV3 Tiny [28] to develop a two-training process. Here, CBAM attention [41] is used to detect large targets; later, a fine-tuning is made to detect small targets.

Recently, advanced versions of the YOLO framework were introduced for ship detection. Zhang_2021 [42] used YOLOV4 with a Reverse Depthwise Separable Convolution (RDSC) to detect ships. The proposed RDSC replaced the Depthwise Separable Convolution (DSC) [43] in the ResUnit of the YOLOV4 backbone. With the help of RDSC, the complexity of the network model is reduced while ensuring accuracy. Han_2021 [44] also uses the YOLOV4 backbone with an attention mechanism to improve performance. Light_SDNet [21] modified the YOLO5 backbone by a Gost Unit [45] and DepthWise Convolution (DWConv) [46] to reduce the number of parameters; also, data augmentations like haze generation and rain generation have been introduced to enrich the training set. Recently, YOLOX has been considered a robust and powerful method for object detection; Zhang_2022 [47] used the YOLOX framework to design a lightweight method. Instead of using a PANnet [48] for feature fusion, the paper used a Lightweight Adaptive Channel Feature Fusion (LACFF) to overcome the inconsistent scale of feature maps. The features of all other layers are adjusted to the same shape. Afterward, the channels are fused according to the learned weights. Similar to Zhang_2022 [47], our work is also based on YOLOX; however, we do not focus on feature fusion but introduce a loss that selects suitable features on the classification head.

Transformer-based methods [13] are also a possible solution for ship detection. Yani_2022 [49] used distillation learning to train a DETR-based ship detector. A teacher model was trained based on a large-scale CoCo Dataset, and the student model was fine-tuned based on the Seaship dataset [19]. The method helps to reduce the FLOPs and number of parameters. However, its mAP is not improved compared with the conventional DETR framework.

## 3. Proposed Method

### 3.1. Overview System

**Figure 2:**
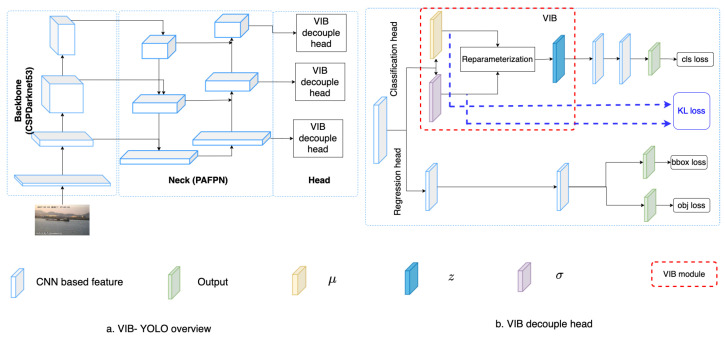
Network structure. (**a**) The overview of proposed VIB-based object detection. (**b**) The proposed VIB-based classification head.

Table 1 summarizes mathematic notations in the paper, and Figure 2 introduces the concept of the proposed method. Given a backbone, features at different scales are extracted. Here, we use the Darknet53 backbone [28] to extract features at multiple scales. The PAFPN [48] serves as a neck that connects these features to a decoupled head. Detail of the backbone and the neck are introduced in Section 3.3. The decouple head includes a classification head and a regression head. While the classification head aims to classify a ship category, the regression head estimates a relative bounding box and the object ability for each cell. On the classification branch, we use 1∗1 kernels to extract the $\mu_{j}\in R^{d}$ and $\sigma_{j}\in R^{d}$ at the $j^{th}$ position on a feature map. Using these kernels, tensors $\mu \in R^{dxHxW}$ and $\sigma \in R^{dxHxW}$ are obtained. These tensors are used to estimate the VIB loss [22]; additionally, a reparameterization process samples a new latent $z_{j}\in R^{d}$ for the $j^{th}$ position. A classifier takes the latent $z\in R^{dHW}$ and predicts the vessel category ${\hat{y}}_{cls}\in R^{KHW}$. The detail of the VIB module is described in Table 2. The kernel size is (1,1) means that the $Encoder_{\mu }$ extracts cross exchange-feature but does not change the size of feature maps. It allows us to reuse the original classification head.

A YOLO detector [11] addresses the bounding box regression, object classification, and category classification at the same time. In our work, the IoU loss ($L_{box}\left({\hat{y}}_{box},y_{box}\right)$) helps to train the bounding box regression, and the cross-entropy loss helps to train the object classification and the category classification. In addition, the VIB loss also helps to select features by introducing a feature selection loss $L_{KL}\left(\mu ,\sigma \right)$. $\alpha_{box}$, $\alpha_{obj}$, $\alpha_{cls}$, $\alpha_{KL}$ are hyper-parameters that control the contribution of $L_{box}\left({\hat{y}}_{box},y_{box}\right)$, $L_{obj}\left({\hat{y}}_{obj},y_{obj}\right)$, $L_{cls}\left({\hat{y}}_{cls},y_{cls}\right)$, and $L_{KL}\left(\mu ,\sigma \right)$, respectively, the loss function in Equation (1) is used to train the detector.

(1) $$L\left(\hat{y},y\right)=\alpha_{box}L_{box}\left({\hat{y}}_{box},y_{box}\right)+\alpha_{obj}L_{obj}\left({\hat{y}}_{obj},y_{obj}\right)+\alpha_{cls}L_{cls}\left({\hat{y}}_{cls},y_{cls}\right)+\alpha_{KL}L_{KL}\left(\mu ,\sigma \right).$$

Recently, the IoU loss ($L_{box}$) has been recognized by many researchers as a good solution to evaluate a predicted bounding box. The IoU loss helps the model to improve the quality of its bounding box predictions by penalizing boxes that do not closely match the ground truth in terms of overlap. It is crucial for achieving accurate object localization in object detection tasks. Equation (2) explains the concept of $L_{\mathrm{IoU}}$.

(2) $$L_{\mathrm{IoU}}\left({\hat{y}}_{box},y_{box}\right)=\sum_{j=1}^{WH}w_{j}^{box}\left(1-IoU_{j}\right)$$

where:

- W,H is the width and height of the output.
- $IoU_{j}$ is intersection over union between the predicted box ${\hat{y}}_{box}$ and the ground-truth box $y_{box}$ at the position $j^{th}$.
- $w_{j}^{box}$ is a mask that decides which locations will be used to compute the loss.

Object classification loss ($L_{obj}\left({\hat{y}}_{obj},y_{obj}\right)$) is concerned with identifying whether there is any object present within a bounding box. It is a binary classification as in Equation (3), where the model predicts a probability score indicating whether an object is present or not in each bounding box. Category classification loss ($L_{cls}\left({\hat{y}}_{cls},y_{cls}\right)$) is focused on determining the specific class or category of the object if one is found. Category classification is a multi-class classification problem, where the model predicts the probability distribution over different object categories for each bounding box as in Equation (4).

(3) $$L_{obj}\left({\hat{y}}_{obj},y_{obj}\right)=\sum_{j=1}^{WH}w_{j}^{obj}\left(t_{j}\cdot \log\left(p_{j}\right)+\left(1-t_{j}\right)\cdot \log\left(1-p_{j}\right)\right)$$

where:

- $p_{j}$ is the predicted objectness probability.
- $t_{j}$ is the ground-truth objectness label.
- $w_{j}^{obj}$ is a mask that decides which locations will be used to compute the loss.

(4) $$L_{cls}\left({\hat{y}}_{cls},y_{cls}\right)=\sum_{j=1}^{WH}w_{j}^{cls}\sum_{k=1}^{C}t_{jk}\cdot \log\left(p_{jk}\right)$$

where:

- *C* is the number of object classes.
- $p_{jc}$ is the predicted class probability (usually obtained through softmax activation).
- $t_{jc}$ is the ground-truth class label for the j-th location and the c-th class.
- $w_{j}^{cls}$ is a mask that decides which locations will be used to compute the loss.

Finally, the feature selection loss is shown in Equation (5). A detailed explanation of the loss will be introduced in Section 3.2.

(5) $$L_{KL}\left(\mu ,\sigma \right)=KL\left(p(z|x)||q(z)\right)=\frac{1}{d}\sum_{j=1}^{WH}\sum_{k=1}^{d}\left(\mu_{j,k}^{2}+\sigma_{j,k}^{2}-2log\left(\sigma_{j,k}\right)-1\right).$$

where:

- *d* is the dimension of latent features.

### 3.2. Feature Section Loss

Feature selection involves the process of choosing pertinent features tailored to a particular task. Drawing from the principles of information bottleneck theory [50], optimal features are concise representations that contain precisely the necessary information to address the task without redundancy. The necessity for this can be elucidated through the following two constraints:

- The latent *z* must help to well predict the output *y* (vessel categories);
- Given the latent *z*, we cannot infer input *x* very well.

In the realms of probability theory and information theory, the interrelation between two variables finds measurement through mutual information (I(.)) [23]. Consequently, these dual constraints are formulated by maximizing the mutual information I(y;z) while minimizing the mutual information I(x;z). The former constraint signifies that *z* aids in predicting vessel categories *y*, while the latter constraint signifies that *z* does not possess the capability to deduce the input image *x*.

Let β represent a Lagrange multiplier; the optimization problem is depicted in Equation (6). A better solution makes $L_{IB}$ have a greater value.

(6) $$L_{IB}=I\left(y;z\right)-\beta I\left(x;z\right).$$

The mutual information I(.) [23] can gauge the information of one variable in relation to another variable by using Equation (7).

(7) $$\begin{array}{cc} I(X;Y) & =I(Y;X)\\ \; & =H(X,Y)-H(X|Y)-H(Y|X)\\ \; & =H(X,Y)-H(X|Y)-H(Y|X)\\ \; & =KL{\left(p(x,y)||p(x)p(y)\right)}_{x\in X,y\in Y}\\ \; & =E_{\left(x,y)∼p(x,y\right)}\left[\log\frac{p(x,y)}{p(x)p(y)}\right]\\ \; & =\int p\left(x,y\right)\log\frac{p(x,y)}{p(x)p(y)}dxdy. \end{array}$$

Thus, I(y;z) and I(x;z) are expressed in Equation (8) and Equation (9), respectively.

(8) $$\begin{array}{cc} I(y;z) & =\int p\left(y,z\right)\log\frac{p(y,z)}{p(y)p(z)}dydz=\int p\left(y,z\right)\log\frac{p(y|z)}{p(y)}dydz\\ \; & =\int p(y,z)\log p(y|z)dydz-\int p(y)\log p(y)dy. \end{array}$$

(9) $$I\left(x;z\right)=\int p\left(x,z\right)\log\frac{p(x,z)}{p(x)p(z)}dxdz=\int p\left(x,z\right)\log\frac{p(z|x)}{p(z)}dxdz.$$

To maximize the mutual information I(y;z), we approximate this term by a lower bound. When the lower bound obtains a greater value, the I(y;z) has a greater value. q(y∣z) is a variational approximation of p(y∣z); the lower bound is founded by Kullback–Leibler divergence as in Equation (10).

(10) $$KL\left(p(y|z)||q(y|z)\right)\geq 0⟹\int p\left(y|z\right)\log p\left(y|z\right)dy\geq \int p\left(y|z\right)\log q\left(y|z\right)dy.$$

By incorporating the lower bound from Equation (10), the expression for I(y;z) in Equation (8) can be reformulated as Equation (11).

(11) I(y;z)≥∫p(y,z)logq(y∣z)dydz−∫p(y)logp(y)dy

In this context, the entropy of the labels H(y)=−∫p(y)logp(y)dy is considered independent and can be disregarded. As a result, the maximum value of I(y;z) is approximated as shown in Equation (12).

(12) $$\begin{array}{cc} I(y;z) & \approx \int p(z|x)p(y|x)p(x)\log q(y|z)dxdydz\\ \; & \approx \int p(z|x)p(y,x)\log q(y|z)dxdydz. \end{array}$$

To minimize the mutual information I(x;z), we approximate this term (Equation (9)) by an upper bound. When the upper bound obtains a smaller value, the I(x;z) has a smaller value. We denote q(z) as a variational approximation to the marginal p(z). Using the KL divergence, the upper bound of I(x;z) is introduced as Equation (13).

(13) $$KL\left(p(z)||q(z)\right)\geq 0⟹\int p\left(z\right)\log p\left(z\right)dz\geq \int p\left(z\right)\log q\left(z\right)dz.$$

Utilizing the upper bound in Equation (9), we can re-express I(x;z) as presented in Equation (14).

(14) $$\begin{array}{cc} I(x;z) & =\int p(x,z)\log p(z|x)dxdz-\int p(z)\log p(z)dz\\ \; & \leq \int p(x,z)\log p(z|x)dxdz-\int p(z)\log q(z)dz\\ \; & =\int p\left(x\right)p\left(z|x\right)\log\frac{p(z|x)}{q(z)}dxdz\\ \; & =\int p\left(x,y\right)p\left(z|x\right)\log\frac{p(z|x)}{q(z)}dxdzdy. \end{array}$$

Through the utilization of the lower bound for I(y;z) and the upper bound for I(x;z), the Lagrangian function in Equation (6) can be approximated as represented in Equation (15).

(15) $$\begin{array}{cc} L_{IB} & =I(y;z)-\beta I(x;z)\\ \; & \approx \int p\left(z|x\right)p\left(y,x\right)\log q\left(y|z\right)dxdydz-\beta \int p\left(z|x\right)p\left(x,y\right)KL\left(p(z|x)||q(z)\right)dxdydz\\ \; & =E_{\left(x,y)∼p(x,y),z∼p(z|x\right)}\left[\log q\left(y|z\right)-\beta KL\left(p(z|x)||q(z)\right)\right]. \end{array}$$

In our application, the term q(y∣z) is modeled by a classifier; and logq(y∣z) is a classification loss $L_{cls}\left({\hat{y}}_{cls},y_{cls}\right)$. In addition, the latent *z* could be sampled from a reparameterization trick g(ϵ,x) where ϵ∼p(ϵ)=N(0,I). Hence, *z* is estimated by Equation (16).

(16) z=μ+ϵ∗σ.

Using Equation (16), the term p(z∣x) is estimated by Equation (17). q(z)=N(0,I), and the term $KL\left(p(z|x)||q(z)\right)$ is estimated by Equation (5). In addition, the term $KL\left(p(z|x)||q(z)\right)$ could serve as a feature selection loss $L_{KL}\left(\mu ,\sigma \right)$ in Equation (1). Hence, the parameter β is replaced by the parameter $\alpha_{KL}$. Equation (5) represents $L_{KL}\left(\mu ,\sigma \right)$ and it is applied at every scale level with classification loss, box loss, and object loss.

(17) $$p\left(z|x\right)=N\left(\mu ,\sigma^{2}\right),$$

### 3.3. Backbone and Neck Module

The proposed method could be integrated with any backbone. However, a modification is needed on the neck to match the selected backbone and the decoupled head. We have tried several backbones in Section 4.5 and pointed out that the Darknet backbone and PAFPN neck can perform better than others.

**Figure 3:**
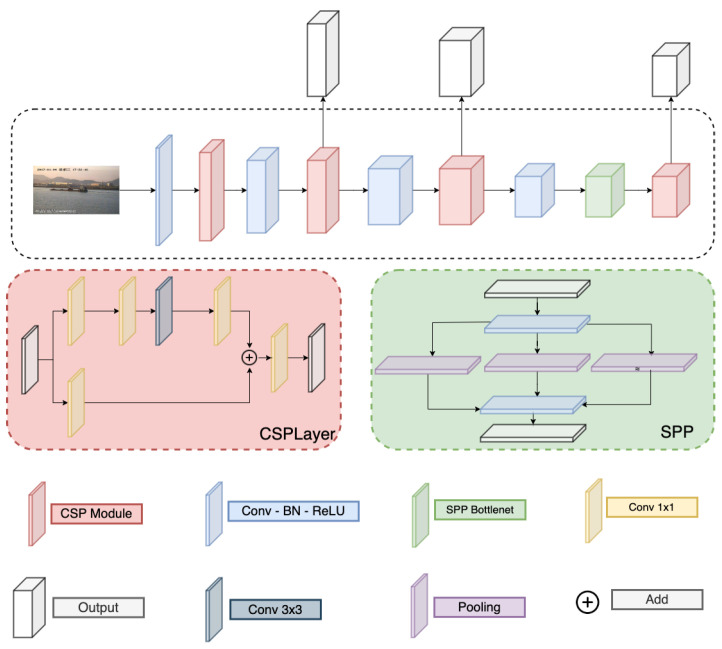
Darknet structure.

**Figure 4:**
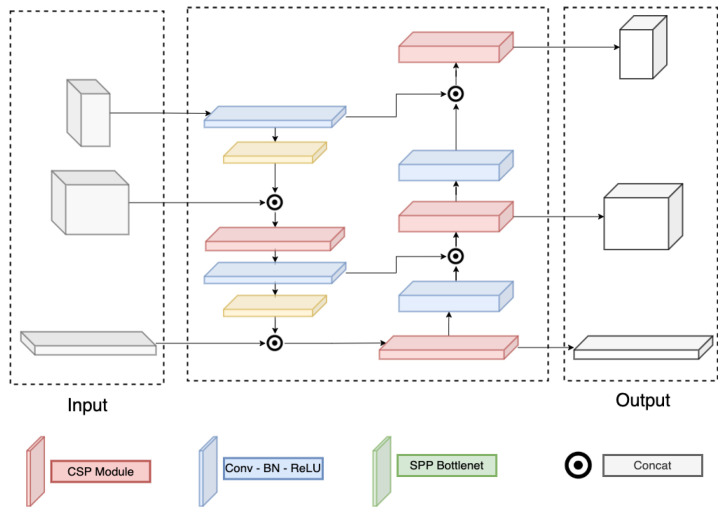
PAFPN structure.

Detail of the Darknet and PAFPN are correspondingly in Figure 3 and Figure 4. Here, the Darknet backbone used CSPLayer to extract features. The features at 2nd, 3rd, and 4th CSPlayer are used in the PAFPN neck. Finally, output features are used with decoupled heads at different scales.

## 4. Experimental Results

### 4.1. Datasets and Experiment Setting

This paper evaluates our proposed method using the SeaShips dataset [19]. The SeaShips dataset is built based on the images captured by an in-field video monitoring system deployed around Hengqin Island, Zhuhai City, China. Each camera records the scene from 6:00 a.m. to 8:00 p.m. every day. The images are obtained from one-minute video clips, resulting in 60 clips per hour. Each clip spans 60 s and contains approximately 1500 frames. For every 50 frames (approximately every two seconds), one image is extracted for inclusion in the dataset. This dataset encompasses six distinct ship types, namely bulk carriers, ore carriers, ordinary cargo ships, container ships, passenger ships, and fishing boats. It features a diverse background environment, characterized by varying light intensities to ensure dataset diversity. Additionally, the dataset incorporates numerous special scenarios, including instances of target occlusions. The SeaShips dataset is highly regarded within the research community for its professionalism, public availability, large-scale nature, and accuracy in ship labeling. It has earned a strong reputation for facilitating effective ship inspection procedures. While dataset [19] has 31,455 images, only 7000 images are published for research at [21,38]. Hence, we use the published dataset for our experiments.

Literature reviews show that many research works use 80% of the published data for training/validating and 20% for testing. Hence, we select $D_{1}^{Train}$, which includes 5600 images for training, and $D_{1}^{Test}$, which includes 1400 images for testing. In addition, recent works [37,49] also use a more challenging setting where 50% of the data are the training set, and the rest is the testing set. We also follow this setting to prepare $D_{2}^{Train}$ and $D_{2}^{Test}$ for comparison. To evaluate the performance on a very small dataset, we randomly select several subsets $S_{1},S_{2},S_{3}$ that include 30%, 70%, and 100% of samples from $D_{2}^{Train}$ for training in later experiments.

Our experiment uses SGD optimizer, learning rate = 0.01, weight decay = 0.001, n_epoch = 200, and batch_size = 8. We use the reduce-mean operator on batch_size, and the reduce-sum operator on prediction output. The mAP is used to select the best model. The loss function in Equation (1) use $\alpha_{box}=10.0,\alpha_{obj=}1.0,\alpha_{cls}=1.0$ and the $\alpha_{KL}=0.125$.

### 4.2. Select the Hyper-Parameter

**Figure 5:**
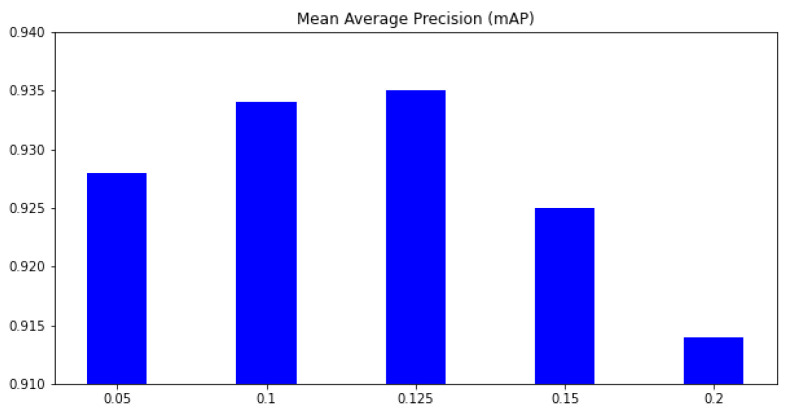
The mean average precision (mAP) on different $\alpha_{VIB}$. *x*-axis means the $\alpha_{KL}$ parameter, and *y*-axis is the mean average precision (mAP) over all classes.

This section aims to select suitable hyperparameters for our training process. The major contribution of our work is introducing the feature selection loss $L_{KL}\left(\mu ,\sigma \right)$ to the YoloX framework. Therefore, our first experiment is selecting a suitable hyper-parameter $\alpha_{KL}$ in Equation (1). A small $\alpha_{KL}$ may not help to learn better features, whereas a large $\alpha_{KL}$ may focus too much on feature learning and forget the main task. In this experiment, the $S_{2}$ dataset is used for training, and the $D_{2}^{Test}$ dataset is used for testing. The mAP metric on six classes is used for comparison. Figure 5 shows how the KL loss affects the result. Without the feature selection, the mAP is only 0.923; when $\alpha_{KL}$ is 0.05, the mAP increases to 0.928. The higher the $\alpha_{KL}$, the higher our mAP. However, when $\alpha_{KL}=0.15$, the mAP begins to be reduced; and if $\alpha_{KL}=0.2$ the mAP is 0.914. The mAP based on $\alpha_{KL}=0.2$ is smaller than when the mAP without feature loss. This phenomenon is because the feature loss reduces the features selected for the main task. When the feature is reduced too much, the classifier may not have enough information for the classification task. In the next experiments, we select the $\alpha_{KL}=0.125$ for our proposed method.

The next experiment aims to evaluate the contribution of other hyperparameters on the performance. In Equation (1), a hyper-parameter controls the contribution of its corresponding loss to the training process. Therefore, a greater hyperparameter will force the algorithm to learn this task first. By adjusting the hyper-parameter settings, we can control the steps of the learning process. For instance, if we want to train the category classification task before other tasks, the hyper-parameter setting should be $\alpha_{cls}=10$ and $\alpha_{box}=\alpha_{obj}=1$. Table 3 shows all settings in detail. The $\alpha_{KL}$ hyperparameter is not included in this table because the feature selection task is an auxiliary task and must be learned lastly after other tasks. By default, we set $\alpha_{KL}=0.125$.

A comparison between the three scenarios is presented in Table 4. Here, the $D_{Train}^{1}$ dataset is used for training, and the $D_{Test}^{1}$ dataset is used for testing. The results show that changing steps of the learning process may not affect the performance too much. Because all component losses are combined into a unique loss by Equation (1), the algorithm will automatically focus on the task that may not work well and ensure all tasks can be learned at the end of a training process. However, in object detection, category classification is conditional on a predicted bounding box. Therefore, from literature reviews, a greater $\alpha_{box}$ can provide better performance. As shown in Table 4, the mAP is 0.989 if we focus on $L_{box}$ first. This value is slightly greater than the performance by learning $L_{obj}$ or $L_{cls}$ first.

### 4.3. Compare with SoTA

This section compares our proposed method with SoTA on the mAP metric. There are several experiment settings from different works. Given 70,000 published images from SeaShip [19], Zhang_2022 [47], and Zhang_2021 [42] used 90% of data for training and validating; the other 10% of data are the testing dataset. Liu_2020 [39], Liu_2022 [36], Han_2021 [44], and Light_SDNet [21] use 80% of data for training and validating dataset, the other 20% is the testing dataset. To compare with these works, we use $D_{1}^{Train}$ for training and $D_{1}^{Test}$ for testing. The result in Table 5 shows that our method is better than other methods in terms of average precision. There are two reasons for this improvement. First, our method is based on the YOLOX framework, which is the recent SoTA frame for object detection. Light_SDNet is based on YOLO5, and its result is also promising. Liu_2020 [39] and Liu_2022 [36] are based on older versions in the YOLO family; hence, the performance is smaller than the SoTA framework as Light_SDNet and our method. Second, the parameterization adds some uncertainty to the training process. It allows the model to work with more data during the training phase. In comparison, Light_SDNet [21] also adds more haze and rain to the original image and gets a very good result (mAP = 0.988%). The major difference between our method and Light_SDNet [21] is how we add noise to training data. While Light_SDNet [21] adds noise to the image domain, our method adds noise to the feature domain. Last but not least, the VIB Loss learns features that focus on the object and remove redundant features in the background. Feature analysis in Section 4.4 will visualize feature maps in detail.

In addition, Biaohua_2022 [37] and Yani_2022 [49] used 50% of published images for training and the rest 50% for testing. Hence, we use $D_{2}^{Train}$ and $D_{2}^{Test}$ for training and testing correspondingly. As shown in Table 5, the mAP on previous works is up to 0.965%. Our proposed method can achieve significantly better performance than previous works. It proves the benefit of our method when the number of training samples is reduced. The primary driving force behind this improvement is the adoption of our method, which builds upon the robust YoloX framework for object detection. It is worth noting that ship detection research typically leverages an object detection framework as its foundation, often with some custom modifications. Therefore, inheriting the capabilities of such a novel and powerful framework naturally leads to improved results.

### 4.4. Contribution of the VIB Loss on Small Datasets

In this section, we discuss the contribution of VIB loss on different scale datasets. The $S_{1}$ (525 images), $S_{2}$ (1225 images), and $S_{3}$ (3500 images) datasets are used for training. The testing dataset $D_{2}^{Test}$ contains 3500 images. The results in Table 6 show that the VIB loss helps improve performance significantly on small datasets. If the training dataset is $S_{1}$, the mAP improves 3%. When the number of training samples is increased, the improvement is reduced. The enhancement on mAP is 1.2% if $S_{2}$ is used for training, and if the training dataset is $S_{3}$, the mAPs from both settings are quite equivalent. This phenomenon is reasonable because an unsupervised loss may help avoid overfitting on small datasets, and a reparameterization allows a classifier to be robust.

To clearly explain the benefit of the proposed method on feature learning, we compare the feature learned by our method (with VIB) and the baseline method (without VIB) using the $S_{3}$ dataset. Features before the last layer of necks and heads are extracted and visualized. We select 20 feature maps with the highest response scores for each scale level. We denote *j* as a pixel on a feature map *F*, which has size (W,H); the score of the feature map is $\frac{1}{WH}\sum_{j=1}^{WH}F_{j}$. These feature maps are accumulated together to formulate a unique response map. The result could represent important pixels on input images.

**Figure 6:**
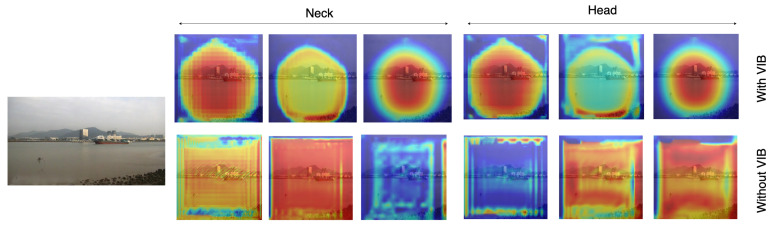
Heatmaps on the classifier head and the neck.

Figure 6 shows the heat map corresponding to an input image. The first row represents feature maps with VIB; the second row represents those without VIB. Since features are extracted at three scale levels, three responses are provided for head modules. The results show that VIB loss can learn features that focus on the object. Without VIB, the response likes a uniform distribution. With VIB, feature responses focus around the object but not all pixels. The phenomenon is also repeated at the neck module. It means the VIB loss can be backpropagated to the neck level and learn a better feature.

In addition, we also evaluate the sparse level and the discriminate level of feature maps. A sparse feature map means many values in the feature map are close to zeros. If a feature map is more sparse, it means the learned filters are not responding to patterns that do not contribute to the prediction process. Also, a sparse feature map means we only select a few features. A discriminate level is the difference between the maximum value and the minimum value in a feature map. A greater discrimination level means some positions have a high response, whereas others have a low response. Hence, the learned filters can strongly respond to useful patterns rather than other patterns. Given a feature map $F\in R^{WH}$ where *j* is a position on the map, the sparse level is estimated by $\sum_{j=0}^{WH}\left|F_{j}<thre\right|$, and the discriminate level is max(F)−min(F).

Utilizing the input image depicted in Figure 6, we extract feature maps from the final layer of the classification head. The statistics regarding the sparsity and discriminative characteristics of these feature maps are presented in Table 7. The findings indicate that VIB generates feature maps with increased sparsity. This outcome can be attributed to the influence of the object function $L_{KL}\left(\mu ,\sigma \right)$, which enforces a zero mean on the features. Furthermore, the higher discriminative level observed in the VIB-based results underscores the robustness of the learned features.

Behind the reasoning experiments, the computational cost has been introduced. Table 8 represents the frame per second (FPS), the Giga-Floating-Point Operations Per Second (GFlops), and the number of parameters of the model. Among them, FPS measures the speed of the model, GFlops evaluates the performance of hardware when running deep learning workloads, and the number of parameters represents the size of the model. The result in Table 8 shows that adding VIB to the network does not increase the computational cost too much. The number of parameters is slightly increased because the VIB module has been added to the network. However, the FPS and GFlops are quite similar in both cases. This means the additional VIB module did not increase the computational cost.

### 4.5. Effect of Backbone

This section discusses how the proposed method works with different backbones. ResNet, MobileNetv2, and DarkNet are used as backbones for comparison. The input channel of the necked is adapted to meet the output of these backbones. The average precisions (AP) for six classes are shown in Table 9. In this experiment, $S_{2}$ serves as a training dataset. The result shows that DarkNet is the best backbone among these pre-trained models. This is reasonable because DarkNet had been recognized as the best backbone in the YOLO family. In addition, the increment given by VIB loss on ResNet [51] is 5.9 %. It means VIB loss can help a lot with some particular backbone.

### 4.6. Effect of Pre-Processing Methods

Our approach enhances the classifier head by introducing a degree of uncertainty to the extracted features. However, it is worth noting that introducing uncertainty into the image domain has been explored in previous works [52,53,54]. For instance, in the Seg-based method [52], researchers trained their model using segmentation images. We have incorporated a similar approach into our ship dataset, generating segmentation images to create a new dataset for training our ship detector. Additionally, the NoiBased approach [53,54] method enriches datasets by introducing noise to input images and employing denoising techniques to bolster system robustness. Drawing inspiration from this observation, we conducted training sessions for our ship dataset both with and without the introduction of noise.

In this context, the $S_{2}$ dataset serves as the training dataset, while $D_{2}^{Test}$ is designated as the testing dataset. The results presented in Table 10 reveal that applying a thresholding method for preprocessing the ship dataset may not yield optimal outcomes. The mean Average Precision (mAP) generated by this method falls short of the results achieved by alternative approaches. This disparity can be attributed to the inherent complexity of cluttered backgrounds within the dataset, making it challenging to identify a single segmentation method suitable for all images. Furthermore, in some instances, portions of ships may inadvertently be excluded, thus compromising the model’s overall performance.

The NoiBased method [53,54] offers a potential remedy by augmenting the dataset through the introduction of noise into the input images. This augmentation leads to a modest performance improvement. Specifically, the mAP registers at 0.923 without the addition of noise, and it increases marginally to 0.925 when noise is incorporated. However, the incremental improvement is relatively slight, possibly because the feature extractor has already learned to filter out noise from the input images, resulting in similarities between the extracted features in both scenarios.

Our approach stands out as the most effective due to the deliberate introduction of uncertainty at the classifier head. In this configuration, the feature extractor is unable to eliminate the introduced uncertainty, placing a greater onus on the classifier to exhibit robustness in handling this uncertainty. This rationale has motivated the incorporation of uncertainty into the feature domain, a practice widely adopted in numerous research studies to enhance model performance.

### 4.7. The Position of VIB Network

**Figure 7:**
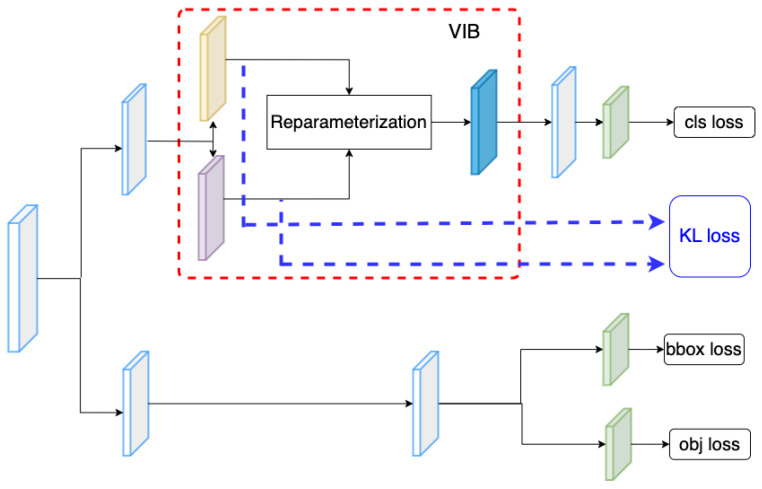
VIB on the mid of classification head.

**Figure 8:**
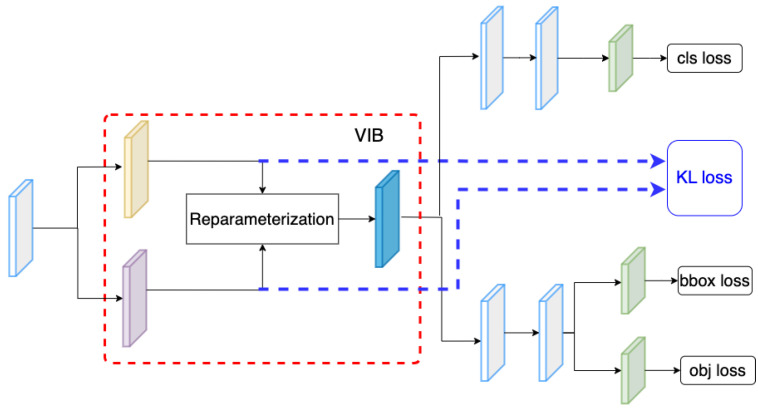
VIB at the beginning of the decouple head.

In the proposed method, we have inserted the VIB module at the beginning of the classification head. However, the VIB model could be inserted at any position of the network structure. Hence, in this section, we have tried several setups to evaluate how to use a VIB in an object detection task. In YOLOX, the classification head has two sequence convolution blocks. The proposed method inserts the VIB block at the beginning of the classification head, as in Figure 2. However, we can also set up the VIB module at the middle of the classification head as in Figure 7 or at the beginning of the decouple head as in Figure 8. Here, the $S_{2}$ dataset is used for training as in Section 4.5. The result in Table 11 shows that the VIB module is only suitable to be inserted on the classification branch. If the VIB module affects the regression branch, the network cannot converge.

**Figure 9:**
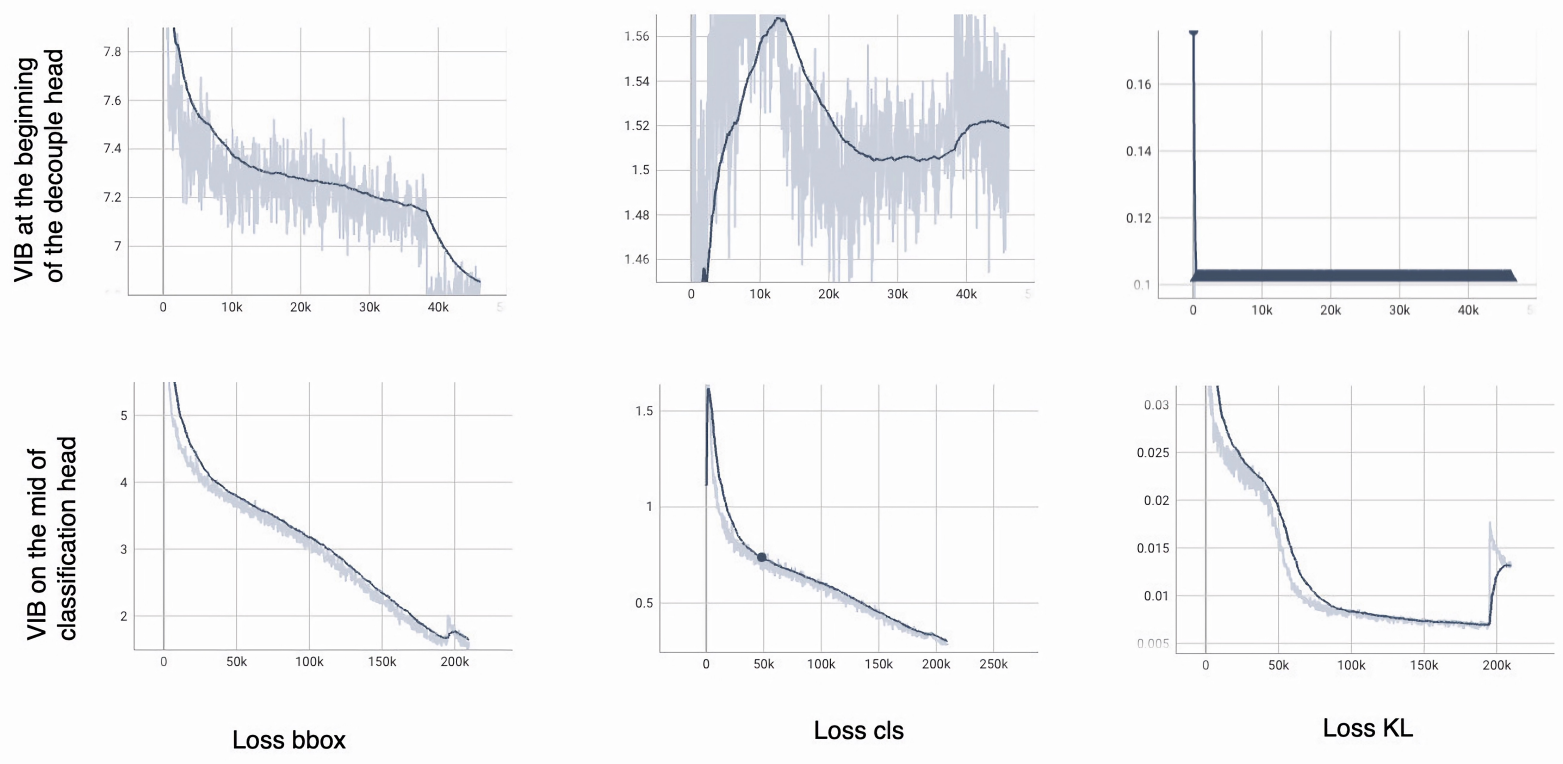
The loss over a training process. The bold line is smooth values over iterations. The light line is the actual value in an iteration.

To explain the phenomenon, the $L_{box}$, $L_{cls}$, and $L_{KL}$ over a training phase are shown in Figure 9.

The model can converge smoothly if the VIB module is on the classification head (the second row of Figure 9). The $L_{box}$ quickly degrades to range [4–5] with only 3000 iterations. Box prediction’s success is a critical requirement to train the classification head. At the beginning of the training process, $L_{cls}$ increases when $L_{box}$ is large; then, it degrades smoothly when the $L_{box}$ is smaller. The $L_{KL}$ should contribute later in the training process because it is an auxiliary loss but not a major task.

The model cannot converge if the VIB module is at the beginning of the decoupled head (the first row of Figure 9). The $L_{box}$ degrades but is still higher than 7 after 40,000 iterations. While the box prediction is unsuccessful, the classification head may be unable to learn. The $L_{cls}$ increases and reduces over a training process. The classification head cannot be learned if $L_{box}$ is still large. This phenomenon shows that the KL loss and the reparameterization make the regression more challenging. Consequently, the classification head cannot be learned, and the model fails to converge.

## 5. Conclusions

In this paper, we proposed a novel method for ship detection. Based on the YOLOX framework, we introduce a VIB module on the classification head of the network. Comprehensive experiments prove that our method is beneficial on small training datasets. The learned features will focus on the object rather than distribute uniformly over images. Our method also provides promising results in comparison with SoTA ship detection.

## Figures and Tables

**Table 1:** Mathematical Notation..

Notation	Description
x,y,z	The input, the output, and the latent feature of the network.
*i*	The index of scale level.
*j*	The index of position on a feature map.
*d*	The dimension of latent vectors in VIB module
$\mu^{i}\in R^{dH^{i}W^{i}},\sigma^{i}\in R^{dH^{i}W^{i}}$	The latent feature and its corresponding variance at the *i*th scale.
$\mu_{j}\in R^{d},\sigma_{j}\in R^{d}$	The feature and its corresponding variance at the position *j*th in a map
$y_{cls}$, ${\hat{y}}_{cls}$	The classification ground truth and output.
$y_{reg}$, ${\hat{y}}_{reg}$	The box ground truth and output.
$y_{object}$, ${\hat{y}}_{object}$	The object ground truth and output.

**Table 2:** Network detail. Here, $Encoder_{\mu }$ means the encoder to extract μ, $Encoder_{\sigma }$ means the encoder to extract σ, *i* is the index of the scale level, and $C^{i}$ is the number of channels in the input of the *i*th level.

Block	Layer	Parameters
$Encoder_{\mu }^{i}$	nn.conv	Size=(1,1), In = $C^{i}$, Out = $C^{i}$
$Encoder_{\sigma }^{i}$	nn.conv	Size=(1,1), In = $C^{i}$, Out = $C^{i}$
Re-parametrize	z=μ+ϵ∗σ	$\epsilon ∼N(0,\sigma^{2})$

**Table 3:** Hyperparameter settings that control the algorithm steps.

Scenario	$\alpha_{\mathrm{box}}$	$\alpha_{\mathrm{obj}}$	$\alpha_{\mathrm{cls}}$
$L_{box}$ first	10	1	1
$L_{obj}$ first	1	10	1
$L_{cls}$ first	1	1	10

**Table 4:** Performance comparisons of hyperparameter settings. The best results are marked in bold.

Scenario	Fishing Boat	Container Ship	Ore Carrier	Bulk Cargo Carrier	Passenger Ship	General Cargo Ship	mAP
$L_{box}$ first	**0.977**	**0.999**	**0.994**	**0.994**	**0.982**	**0.987**	**0.989**
$L_{obj}$ first	0.967	0.987	0.992	0.992	0.942	0.987	0.978
$L_{cls}$ first	0.966	0.988	0.984	0.990	0.959	0.990	0.979

**Table 5:** Performance comparisons of SoTA methods. The best results are marked in bold.

Method	Train + Val/Test (in %)	Fishing Boat	Container Ship	Ore Carrier	Bulk Cargo Carrier	Passenger Ship	General Cargo Ship	mAP
Zhang_2022 [47]	90/10	0.824	0.940	0.859	0.915	0.787	0.914	0.873
Zhang_2021 [42]	90/10	-	-	-	-	-	-	0.946
Liu_2020 [39]	80/20	-	-	-	-	-	-	0.908
Liu_2022 [36]	80/20	-	-	-	-	-	-	0.964
Han_2021 [44]	80/20	-	-	-	-	-	-	0.906
Light_SDNet [21]	80/20	**0.986**	0.995	**0.989**	0.990	0.982	0.989	0.988
Proposed method	80/20	0.979	**1**	0.987	**0.994**	**0.994**	**0.993**	**0.991**
Yani_2022 (ESDT) [49]	50/50	-	-	-	-	-	-	0.593
Yani_2022 (DETR) [49]	50/50	-	-	-	-	-	-	0.965
Biaohua_2022 [37]	50/50	0.940	0.987	0.966	0.978	0.937	0.972	0.963
Proposed method	50/50	0.970	0.986	0.984	0.991	0.964	0.989	**0.98**

**Table 6:** Performance on small datasets. $S_{1}$ means 30% training samples, $S_{2}$ means 70% training samples, $S_{3}$ means 100% training samples from $D_{2}^{Train}$. The best results are marked in bold.

Method	Metrics	Fishing Boat	Container Ship	Ore Carrier	Bulk Cargo Carrier	Passenger Ship	General Cargo Ship	mAP
$S_{1}$ with VIB	dets	2849	1272	5374	3581	774	3598	
	recall	0.878	**0.941**	**0.924**	**0.913**	**0.657**	**0.946**	
	AP	0.796	0.884	**0.831**	**0.765**	**0.524**	**0.794**	**0.766**
$S_{1}$ without VIB	dets	3201	884	4654	2851	696	2944	
	recall	**0.892**	0.920	0.923	0.876	0.637	0.940	
	AP	**0.805**	**0.890**	0.822	0.710	0.466	0.743	0.739
$S_{2}$ with VIB	dets	1863	584	2012	1653	354	1129	
	recall	**0.940**	**0.984**	0.962	**0.971**	0.891	0.964	
	AP	**0.922**	**0.980**	**0.935**	**0.953**	0.873	**0.946**	**0.935**
$S_{2}$ without VIB	dets	2324	674	2848	1923	570	1585	
	recall	0.936	0.975	0.964	**0.963**	**0.899**	**0.978**	
	AP	0.903	0.970	0.932	0.928	0.862	0.945	0.923
$S_{3}$ with VIB	dets	1544	466	1562	1341	297	948	
	recall	**0.978**	0.986	0.990	**0.995**	**0.972**	**0.993**	
	AP	**0.970**	0.986	0.984	**0.991**	**0.964**	**0.989**	**0.98**
$S_{3}$ without VIB	dets	1574	470	1561	1360	294	883	
	recall	0.965	**0.989**	**0.995**	**0.993**	0.960	**0.993**	
	AP	0.957	**0.988**	0.990	0.987	0.953	**0.989**	0.977

**Table 7:** Statistics of sparse level and discriminate level on feature maps.

	With VIB		Without VIB	
	Sparse ↑	Discriminate ↑	Sparse ↑	Discriminate ↑
mean	194.44	32.45	0.183	0.587
std	136	59.557	0.486	0.599
min	0	0.277	0	0.203
percentiles (25%)	0	0.6698	0	0.203
percentiles (50%)	270	0.6698	0	0.3648
percentiles (75%)	304	34.011	0	0.5318
max	335	281.06	3	4.2183

**Table 8:** A comparison between computation cost with and without using VIB.

	With VIB	Without VIB
FPS	12.39	12.45
GFlops	9.92	9.91
# parameters (M)	55.33	54.15

**Table 9:** A mAP comparison with and without VIB on different backbones.

Backbone	With VIB	Fishing Boat	Container Ship	Ore Carrier	Bulk Cargo Carrier	Passenger Ship	General Cargo Ship	mAP
ResNet-18	Yes	0.837	0.978	0.861	0.886	0.741	0.931	**0.873**
	No	0.817	0.961	0.824	0.735	0.706	0.841	0.814
DarkNet	Yes	0.922	0.980	0.935	0.953	0.873	0.946	**0.935**
	No	0.903	0.970	0.932	0.928	0.862	0.945	0.923
MobileNetv2	Yes	0.895	0.975	0.895	0.880	0.794	0.908	**0.891**
	No	0.872	0.965	0.918	0.902	0.772	0.914	0.890

**Table 10:** Performance comparisons among pre-processing methods. The best results are marked in bold.

Method	Fishing Boat	Container Ship	Ore Carrier	Bulk Cargo Carrier	Passenger Ship	General Cargo Ship	mAP
Seg-based [52]	0.873	0.952	0.896	0.859	0.805	0.894	0.880
NoiBased [53,54]	0.912	0.973	0.923	0.935	0.871	0.935	0.925
No noise	0.903	0.970	0.932	0.928	0.862	0.945	0.923
Proposed method	**0.922**	**0.980**	**0.935**	**0.953**	**0.873**	**0.946**	**0.935**

**Table 11:** Performance when the VIB module is inserted at different positions on the YOLOX framework.

Method	Metrics	Fishing Boat	Container Ship	Ore Carrier	Bulk Cargo Carrier	Passenger Ship	General Cargo Ship	mAP
VIB at the middle of classification head	dets	1863	584	2012	1653	354	1129	
	recall	0.940	0.984	0.962	0.971	0.891	0.964	
	AP	0.922	0.980	0.935	0.953	0.873	0.946	0.935
VIB at the beginning of decouple head	dets	-	-	-	-	-	-	
	recall	-	-	-	-	-	-	
	AP	-	-	-	-	-	-	

## Data Availability

The data presented in this study are openly available in [Zhang] at [https://doi.org/10.1109/ACCESS.2022.3199352], reference number [21].
